# Erwin Bünning and Wolfgang Engelmann: establishing the involvement of the circadian clock in photoperiodism

**DOI:** 10.1007/s00359-024-01704-7

**Published:** 2024-05-28

**Authors:** Charlotte Helfrich-Förster

**Affiliations:** Neurobiology and Genetics, Biocentre, University of Würzburg, Würzburg, Germany

**Keywords:** Photoperiodism, Circadian clock, *Kalanchoë blossfeldiana*, *Phaseolus multiflorus*, Erwin Bünning, Wolfgang Engelmann

## Abstract

In 1936, Erwin Bünning published his groundbreaking work that the endogenous clock is used to measure day length for initiating photoperiodic responses. His publication triggered years of controversial debate until it ultimately became the basic axiom of rhythm research and the theoretical pillar of chronobiology. Bünning’s thesis is frequently quoted in the articles in this special issue on the subject of “A clock for all seasons”. However, nowadays only few people know in detail about Bünning’s experiments and almost nobody knows about the contribution of his former doctoral student, Wolfgang Engelmann, to his theory because most work on this topic is published in German. The aim of this review is to give an overview of the most important experiments at that time, including Wolfgang Engelmann’s doctoral thesis, in which he demonstrated the importance of the circadian clock for photoperiodic flower induction in the Flaming Katy, *Kalanchoë blossfeldiana*, but not in the Red Morning Glory, *Ipomoea coccinea*.

## Introduction

In 1936, Erwin Bünning^1^ submitted his groundbreaking work that the endogenous clock is the “Grundlage” (mostly translated as “basis”) for photoperiodic responses. This paper is published in German in the “Berichte der Deutschen Botanischen Gesellschaft” (Reports of the German Botanical Society) (Bünning 1936). Erwin Bünning was 30 years old at that time, and the paper was the result of five years of work at the Universities of Jena and Königsberg. His publication triggered years of controversial debate until it ultimately became the basic axiom of rhythm research and the theoretical pillar of chronobiology. In a retrospective, Bünning (1987) writes: *“This publication was initially met with general rejection at home and abroad. The existence of an endogenous daily rhythm was not generally known or recognized. Some authors even wrote that I had invented such a rhythm in order to explain photoperiodism. The assumption that such rhythms could be used as a clock to measure the length of days was even described as mysticism and metaphysics in extreme cases. If I had been older than 30 at the time, I would not have dared to develop such a hypothesis. In the meantime, circadian rhythm has long since become textbook-ready, and Current Contents in October 1982 wrote in the This Week’s Citation Classic section: “This paper has been cited in over 135 publications since 1961. It proved to be the most-cited paper ever published in this journal”.”* Even more papers (namely 319) cite his summarizing article published in English in the Cold Spring Harbor Symposia on Quantitative Biology (Bünning 1960). In the present special issue, the majority of authors referred to Bünning’s hypothesis that the endogenous clock plays a role in photoperiodic responses (Bradshaw et al. 2023; Colizzi et al. 2023; Fishman and Tauber 2023; Floessner et al. 2023; Hamanaka et al. 2023; Hidalgo and Chiu 2023; Monecke 2023; Oda and Velentinuzzi 2023; Saunders 2023; Schmal 2023; Vaze et al. 2023). Since most of Bünning’s original work on this topic, including the work of his former student Wolfgang Engelmann^2^, has been published in German, I would like to take the opportunity to summarize the most important early results in this review. Wolfgang Engelmann died on July 1, 2023, just three months after his friend David Saunders had passed away (see obituary from Helfrich-Förster this issue). Unlike Saunders, Engelmann was not primarily working on photoperiodic phenomena and insect diapause, but in his doctoral work he performed crucial experiments on the Flaming Katy (*Kalanchoë blossfeldiana*) that strongly support Bünning’s hypothesis, and also on the Red Morning Glory (*Ipomoea coccinea*), where the experimental results did not support Bünning’s hypothesis. Subsequently, Engelmann joined the laboratory of David Shappirio where he demonstrated the photoperiodic control of the larval diapause in the dipterean *Chironomus tentans*. This work led to a well-received publication in *Nature*, in which his last name was misspelled as Englemann (Englemann and Shappirio 1965). After his postdoctoral stay with Colin Pittendrigh^3^, Engelmann combined his experience with plant and animal physiology and biological clocks to study rhythmic phenomena in a large number of different species (Engelmann 2004, 2008, 2009a). Engelmann had a lifelong interest in photoperiodism, and, among his many online books, he wrote one about the “biocalendar” that enables organisms to recognize the season (Engelmann 2009b). This book describes not only the seasonal germination of seeds and the induction of flowering in plants, but also the annual eclosion of dinoflagellates from their cysts, the annual rhythms of body weight, fur color, reproduction, and behavior of the Djungarian hamster, and the annual molt and migration of birds.

Here, only Bünning’s and Engelmann’s initial work will be described. A biography of Bünning’s life and scientific work can be found in Plesse (1996) and a brief overview of Engelmann’s work in Helfrich-Förster (2023).

### Bünning’s characterization of circadian rhythms in bean plants and flies

Bünning started to work on the daily leaf movements of bean plants in 1928, shortly after he had finished the experimental work for his doctoral thesis on the seismoreactions of stamens and stigmas at the University of Berlin. At that time Friedrich Dessauer, the director of the Institute for the Physical Foundations of Medicine in Frankfurt, was looking for two physiologists to carry out research into the possible influence of air ions on plants. Bünning went to Frankfurt together with Kurt Stern^4^ who had at that time already written a book about electrophysiology of plants. At this time, Rose Stoppel^5^ had thoroughly investigated the so-called daily sleep movements of the bean plant *Phaseolus multiflorus* that continued in the absence of daily light-dark and temperature cycles and concluded that there must be an unknown and hypothetical “Factor X” in the air that provokes them (Stoppel 1916, 1920). Bünning and Stern included Stoppel in their studies, and together they searched for this Factor X without success. They finally concluded that the plants’ rhythmic movements are not caused by rhythmic changes in air ions (Bünning et al. 1930).

In the following years, Bünning continued the experiments on the leaf movements of *Phaseolus multiflorus* under continuous light and constant temperature conditions and gained additional experimental evidence that they are independent of environmental 24-h changes (i.e., they display an endogenous periodicity, later called circadian) (Bünning 1931). Bünning was not the first person who noticed that the leaf movements of bean plants continue in *absence* of any environmental Zeitgebers. Wilhelm Pfeffer^6^ described this already in 1907 for *Phaseolus*, but he was not convinced that there exists an endogenous clock because these rhythmic movements cease after several days under constant light (Pfeffer 1907). Anthonia Kleinhoonte^7^ on the other hand described the rhythmic leaf movements for the Jack bean (*Canvalis ensiformis*) in detail in her 1928 doctoral thesis, which she translated into German and published in 1929 and 1932. She concluded from her experiments that the rhythms are endogenous (Kleinhoonte 1932). Nevertheless, Bünning was the first scientist who demonstrated that circadian rhythms are inherited by crossing bean plants with short and long free-running periods (Bünning 1932, 1935a). He also studied rhythms in other organisms, and in 1935, he published his observation that the eclosion rhythms of *Drosophila* also continue under constant conditions (Bünning 1935b). From then on, it was clear to him that plants and animals have endogenous clocks. The question remained as to why they need them, as organisms usually experience daily environmental changes that might be expected to tell them the time.

### Circadian clocks enable organisms to be prepared for seasonal changes

Although Bünning was aware that a circadian clock enables organisms to prepare themselves in advance for the daily rhythmic changes and that they may have an advantage in time memory, which Ingeborg Beling^8^ had just demonstrated in honeybees (Beling 1929), he was not convinced that this represented a sufficient selective advantage to be maintained during evolution. However, from his experiments in bean plants he raised the hypothesis that circadian clocks are essential for measuring day length and preparing in time for the coming season. More specifically, circadian clocks are perfectly suited to provide an internal reference necessary for measuring day length. He reasoned that a failure to prepare in time for the winter will ultimately result in death, while a failure to prepare for reproduction in spring will result in no offspring. Therefore, possessing a circadian clock has a strong selective advantage (Bünning 1932). In the following, I will describe Bünning’s initial experiments from which he concluded the involvement of the circadian clock in measuring day length.

**Fig. 1:**
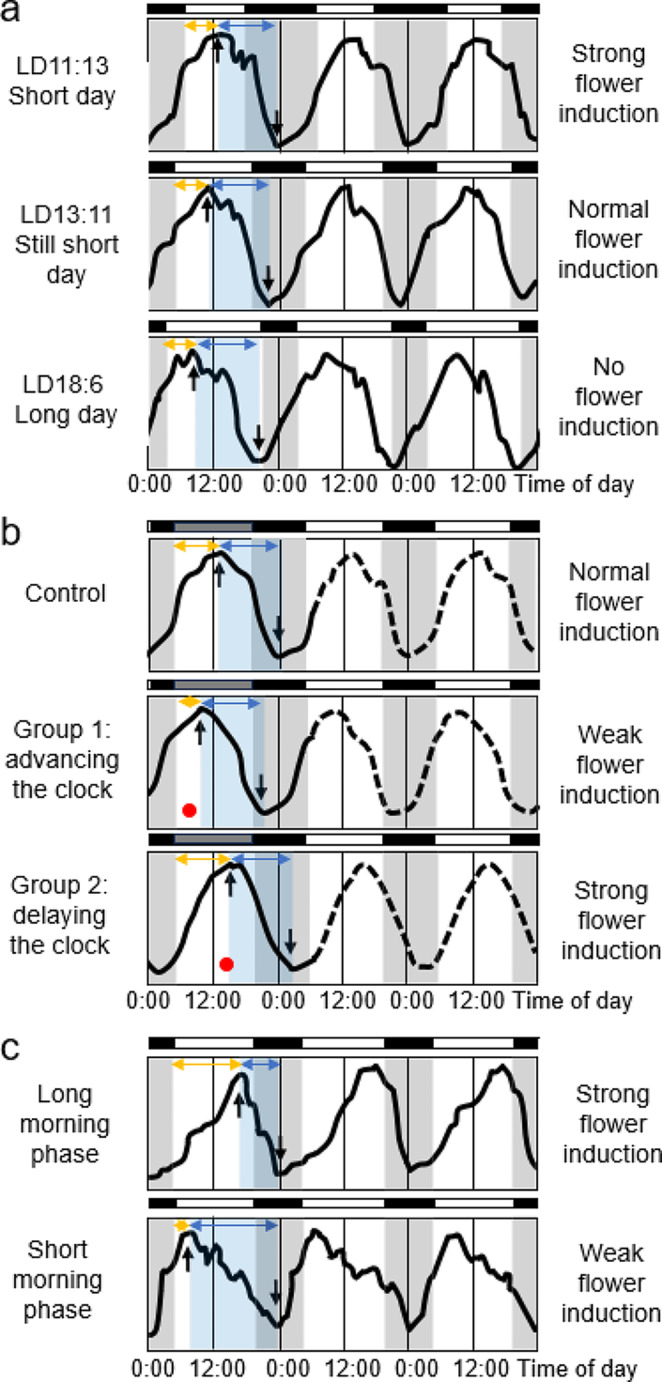
The experiments of Bünning that let him conclude that the circadian clock is involved in photoperiodism. **a** Rhythmic leaf movements of *Phaseolus multiflorus* plants grown in a greenhouse during autumn (October; light-dark cycle (LD)11:13), spring (April, LD13:11) and summer (June, LD18:6). The leaves reached their day-position (arrows up) always 4–6 h after lights-on (orange double-headed arrow) and the night-position (arrows down) 8–10 h later (blue double-headed arrow). This means that a considerable part of the leaves’ evening phase (blue shade) was in darkness (grey shade and black bar on top) in spring and autumn, while the leaves’ evening phase was completely in light in summer. The proportion by which the evening phase was in darkness determined the degree of flower induction. **b** Advancing or delaying the clock by red light given every day for 1 h either in the morning or the afternoon (red dots) changes the number of hours in which the evening phase is in darkness and consequently the induction of flowering. Please note that in this experiment the leaf movements were only monitored during the last day in the greenhouse under constant darkness after the shift was performed and before they were planted in the field (solid lines), where they experienced the still short days of April. The curves were manually continued (broken lines) to illustrate their putative relationships to the environmental LD cycle. **c** Rhythmic leaf movements of two *Phaseolus* plants with long and short morning phases, respectively. The plant with the long morning phase had a short evening phase that was almost completely in darkness under a long day of May, while the other plant had a long evening phase of which only a small portion was in darkness. Consequently, they showed strong and weak flower induction, respectively. Redrawn after Bünning (1936)

By carefully watching the leaf movements of *Phaseolus* in the greenhouse at different seasons Bünning noticed that the shape of the oscillations was always the same: the day-position (leaves-up) was always reached 4–6 h after lights-on and the night-position (leaves-down) 8–10 h later. This means that the evening phase of the leaves (lowering the leaves to its night position) was in darkness in spring and autumn but in light in summer (Bünning 1936). This gave him the idea that the time during which the evening phase is in darkness or light is an indicator of day length (Fig. 1a). Since *Phaseolus* is a short-day plant, flowering would be induced when the evening phase is mostly in darkness, while the plant would remain in the vegetative phase when light in the evening phase exceeds a threshold duration. To test this idea, Bünning decided to shift the circadian clock by exposing seedlings to red light (> 600 nm), to which the clock is very sensitive, and then test the effect on flowering. He sowed the seeds in March in the greenhouse under normal conditions. At a growth of 5–8 cm he transferred the seedlings into a dark chamber and illuminated them with red light for an hour every day. The first group received light from 8:00 to 9:00, the second group from 14:00 to 15:00. Consequently, the endogenous clock was shifted differently in the two groups. In the first group, the leaves had their day position at 8:00 to 9:00 and the night position at 21:00 to 22:00, thus the phase of the oscillation was advanced. In the second group, the leaves had the day position at 14:00 to15:00 and the night position at 2:00 to 4:00 meaning that the oscillation was delayed (Fig. 1b). The seedlings were then transferred to natural conditions in mid of April (still short-day conditions) and the flowering time was determined. The plants of group 1 had now most of their night phase in the light and showed reduced flower induction (they flowered late), while the plants of group 2 had their night phase mostly in the dark and showed a strong flower induction (they flowered early). Bünning then repeated the experiment with beans that he had selected for a short and long morning phase, respectively (Fig. 1c). The plants with long morning phase had almost their entire evening phase in the dark even under the long-day conditions of May and showed a strong flower induction, meaning that they behaved as long-day plants instead of short-day plants. In contrast, the plants with a short morning phase had a considerable part of their evening phase in the light, which only led to a very weak flower induction. These plants needed a very short day to induce flowering. Bünning postulated that the shape of the daily oscillation can determine whether a plant is a short-day or long-day plant. Furthermore, he concluded that the circadian clock is the physiological “basis” of photoperiodism and that it has two different light-sensitive phases, the morning phase during which light promotes flowering (also called photophilic phase) and the evening phase during which light suppresses flowering (also called scotophilic phase) (Bünning 1936).

**Fig. 2:**
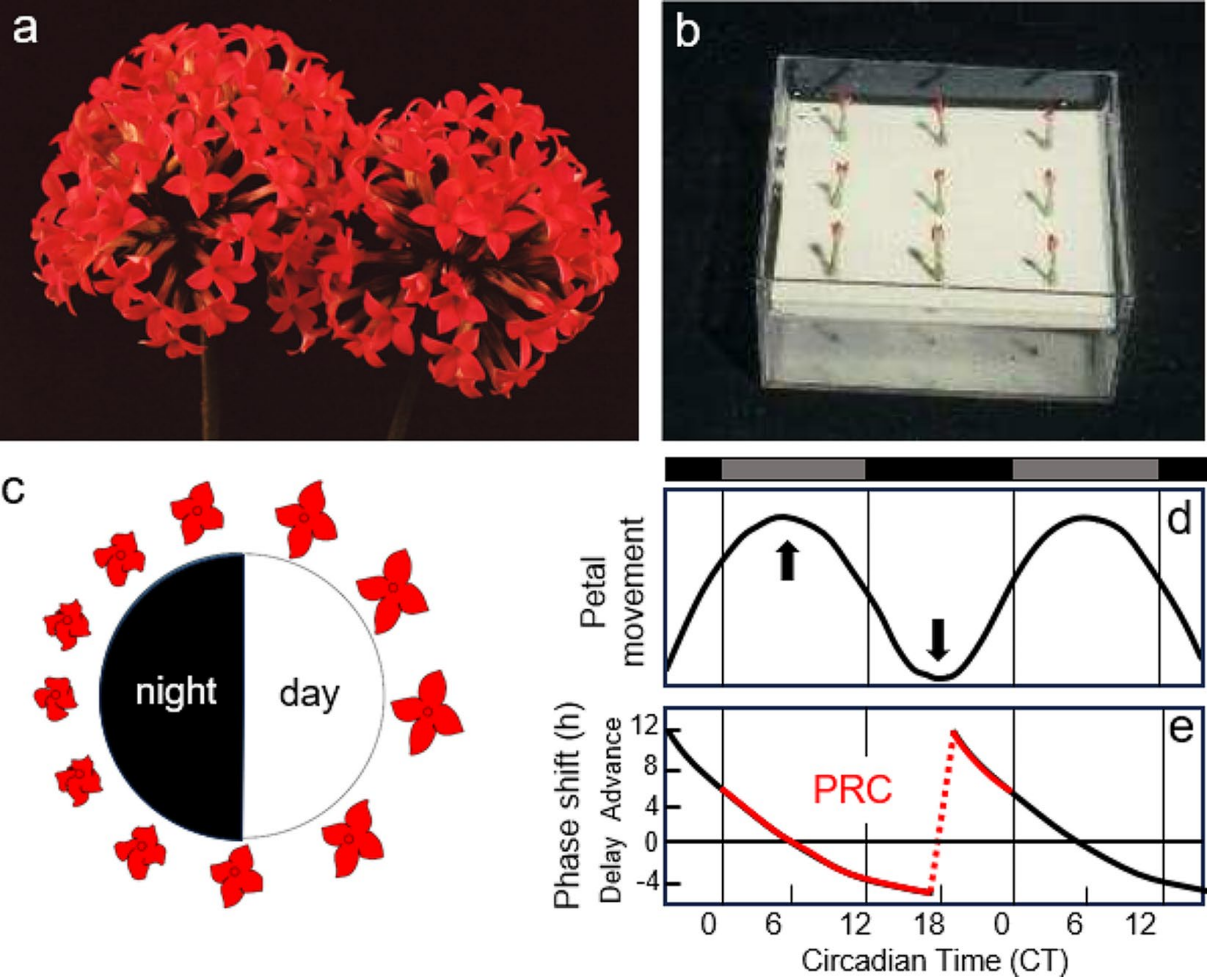
Petal movements of *Kalanchoë blossfeldiana* and the Phase Response Curve to light. **a** Inflorescence of the Flaming Katy. **b** The petal movements can be recorded in individual flowers; here all flowers are in their night state and therefore closed. **c** In diurnal 12:12 h cycles, the flowers are completely opened always near the middle of the light period and completely closed in the middle of the dark period. **d** This petal movement continues under constant darkness. Here, two cycles of petal movement are shown. The previous light periods are indicated as grey bars on top of the diagram. The upward arrow points to the completely opened flower in the middle of the first subjective day, whereas the downward arrow indicates the closed flower in the middle of the subjective night. **e** Phase shifts of the rhythm evoked by 2 h far-red light pulses administered at different times of the subjective 24 h day. The start of the subjective day is at circadian time CT 0, while the beginning of the subjective night is at CT12. Depending on the time of day at which the light pulse was given, the rhythm of petal movement is advanced or delayed (see text). The red part of the curve indicates the conventional Phase Response Curve (PRC) which shows relatively small phase shifts during the subjective day but large phase shifts during the subjective night. a-c: after Engelmann and Antkowiak (2016), d-e: modified after Zimmer (1962)

Indeed, circadian clocks have phases of different light sensitivity, which can be visualized in a so-called Phase Response Curve by giving single light pulses at different times to an organism held under continuous darkness (Johnson 1999). Bean plants cannot be held for a longer time in continuous darkness. Therefore, the responses of light-pulses on rhythmic leaf movements are difficult to assess. More suited for these experiments are rhythms of petal opening and closing such as those of the Flaming Katy (*K. blossfeldiana)*, which continue in the absence of light (Fig. 2). *K. blossfeldiana* is a glabrous, bushy, evergreen, and perennial succulent plant native to Madagascar. It is also known as Christmas *Kalanchoë*, because it belongs to the short-day plants that flower around Christmas time. Its flowers open and close regularly for many days, and this rhythm can be recorded in single flowers isolated from the plant (Fig. 2b-d). During the morning phase (petal opening) light-pulses advance the rhythm, while they delay the rhythm during the evening phase (petal closing) (Zimmer^9^ 1962) (Fig. 2e). During the middle of the subjective night, when the petals are closed, the Phase Response Curve shows a sudden jump from phase delays to advances, which is called “breakpoint”. It is important to note that the extent of delays and advances depends not only on the subjective time of day, but also on the intensity and duration of the light pulses. The longer and more intense the light pulses, the greater the phase shifts. Light pulses in the range of few minutes usually do not cause any phase shifts. This makes the clock robust against random changes in light during the subjective night.

Phase Response Curves to light-pulses of different organisms can be very different (see Carl Johnson’s atlas of Phase Response Curves at https://as.vanderbilt.edu/johnsonlab/prcatlas/), but they have also similar characteristics (Johnson 1992, 1999). Usually, they have phase advances in the late subjective night and early subjective day (from circadian time (CT) ~ 20 to ~ 4), phase delays in the late subjective day and early subjective night (CT ~ 8 to ~ 16), and only small phase shifts in the middle of the subjective day (CT ~ 4 to ~ 8). Many animals have a “dead zone” in the middle of the subjective day, during which the clock is not shifted at all by light-pulses (Daan and Pittendrigh 1976). This dead zone is absent in the Phase Response Curve of *Kalanchoë*, only at CT6 light-pulses cause no phase shifts (Fig. 2e). Nevertheless, even in *Kalanchoë*, light-pulses given during the subjective day cause much smaller phase shifts than those given in the subjective night (Fig. 2e).

**Fig. 3:**
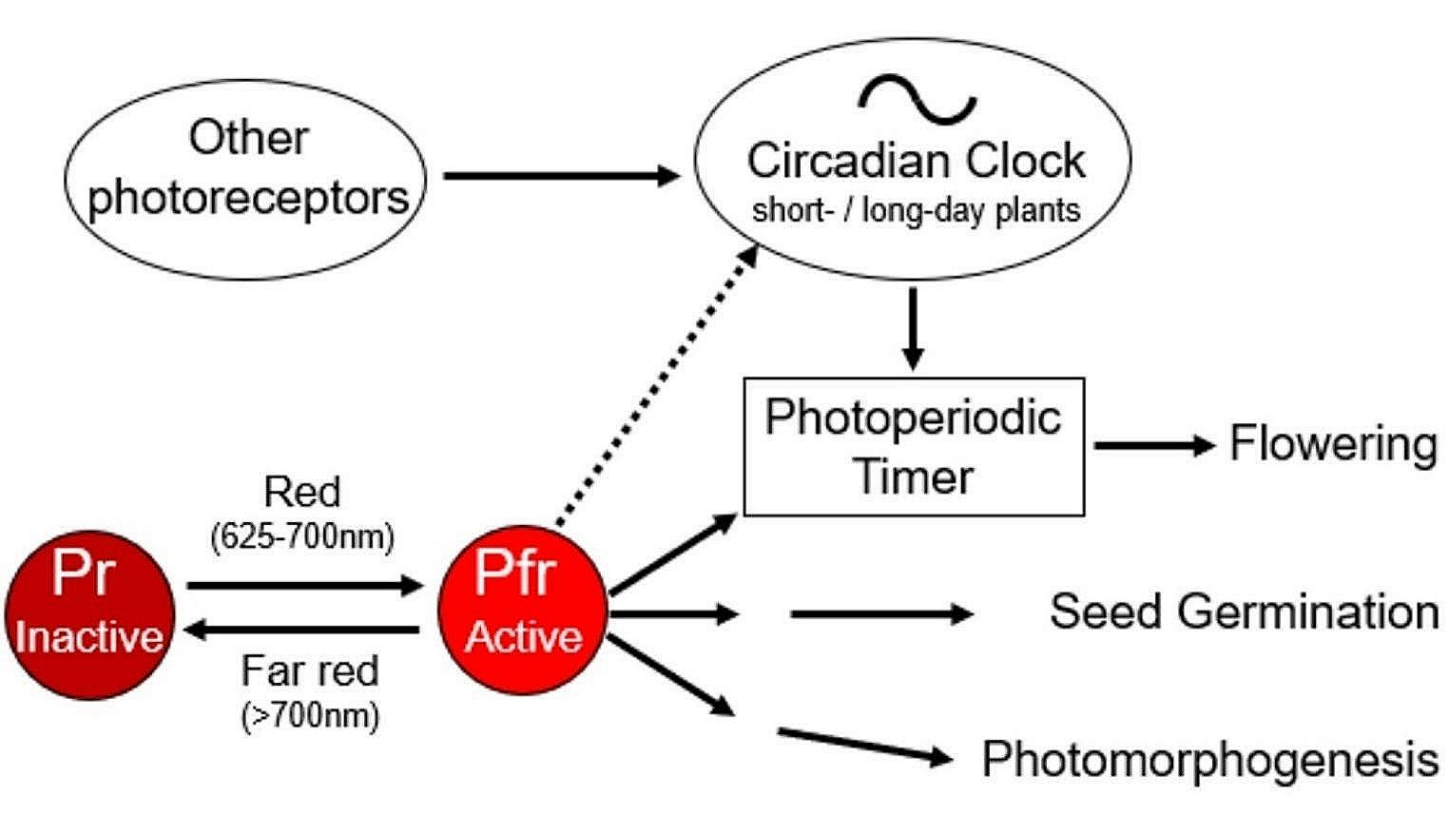
The photoreversible phytochrome system. Pr is the inactive form of phytochrome that is converted into the active Pfr form by red light. Far-red light reverses the active Pfr form back into the inactive Pr form. Pfr signals to the photoperiodic timer and the latter inhibits flowering in short-day plants when it receives simultaneously signals from the circadian clock in its scotophilc phase. In long-day plants the photoperiodic timer needs to receive simultaneously signals from the clock in its photophilic phase. In addition to flowering, the active Pfr form promotes seed germination and photomorphogenesis. Seed germination is seasonally regulated and therefore also needs input from the circadian or the circannual clock (Engelmann 2009b) (not shown here). Pfr signals also to the circadian clock (stippled arrow), but it is not the clock’s only photoreceptor. Short red-light pulses that affect the photoperiodic timer cannot phase-shift the clock

Most importantly, the Phase Response Curve indicates different physiological states of the circadian clock with different light responsiveness. In the case of the short-day plants *Phaseolus* and *Kalanchoë*, light falling into the delay zone (the scotophil phase) appears to inhibit flowering, suggesting that the circadian clock directly inhibits flowering after receiving light in its scotophil phase. However, this is not the case. As we know now, to inhibit flowering, light must hit the so called “photoperiodic timer”, which controls the photoperiodic response by comparing information from the photoperiodic photoreceptors with that from the circadian clock (Saunders 2002; Bradshaw and Holzapfel 2017; see also Colizzi et al. this issue for a definition). The photoreceptors that set the circadian clock are therefore not necessarily the same as those that signal to the photoperiodic timer. Already Bünning wrote in the 3rd edition of his book (Bünning 1973) that the ability of the clock to enforce a rhythm in its reaction to light (e.g. the photoperiodic response) is something quite different from its ability to become entrained by light signals. He explained this by the fact that different photopigments are used and that the corresponding photoreceptors are located in different organs (e.g. in the upper epidermis for photoperiodic reactions and in the leaf joint for rhythmic leaf movements). However, he did not mention the photoperiodic timer as additional element in photoperiodism since nothing was known about its existence when Bünning performed his experiments. Nevertheless, Bünning’s doctoral student Walter Könitz^10^ (1958) carried out decisive experiments with colored light that pointed to the underlying photoreceptors for the photoperiodic response. He found that only red light (~ 660 nm) inhibits flowering in the scotophil phase, whereas far-red light (~ 730 nm) does not have any effect in the scotophil phase, but it inhibits in the photophilic phase. As we know now, this is caused by the photoconvertible phytochrome system (reviewed in Huq et al. 2024). Phytochromes have two forms, the “red form” (Pr) that absorbs red light (625–700 nm) to be converted into the “far-red” form (Pfr), and the Pfr form that absorbs far-red light (> 700 nm) to be converted into Pr (Fig. 3). These two forms of phytochrome can be repeatedly interconverted by light pulses. The “far-red” absorbing Pfr form is the physiologically active form that is thought to repress flowering in short-day plants but promote flowering in long-day plants by acting on the photoperiodic timer (Fig. 3). Pfr is also the phytochrome form that promotes seed germination and photomorphogenesis (reviewed in Huq et al. 2024).

Most importantly, the two phytochrome forms are extremely light sensitive. It was found that as little as one minute of red light is sufficient to trigger germination of lettuce seed but that one minute of far-red light immediately following this can cancel out the red-light effect and thus block germination (Borthwick et al. 1952). The circadian clock is also sensitive to red light, but it needs at least red light-pulses of 1 to 2 h to be phase shifted (see Fig. 2). For normal entrainment to light-dark cycles and a maximum sensitivity to phase-shifting light pulses it uses additionally other photoreceptors that respond also to shorter wavelengths (see also Bünning 1973). This observation indicates that it is not the clock that directly leads to the inhibition of flowering. It is the photoperiodic timer that controls flowering by integrating information from the circadian clock and the phytochrome system (Fig. 3).

The molecular basis of photoperiodic flowering is best understood in the long-day plant *Arabidopsis thaliana* (Salazar et al. 2009; Song et al. 2015; Gendron and Staiger 2023). Here, I will only describe its basic features. *Arabidopis* has five phytochromes, among which the function of PHYA is best described. The active Pfr form of PHYA interacts with CONSTANS, a clock-controlled factor and potent promotor of flowering. CONSTANS is the missing link between the circadian clock and photoperiodic timing (Suárez-López et al. 2001; Yanowsky and Kay 2002). It undergoes clock-controlled oscillations with maxima in the morning and evening (Song et al. 2018). In the dark, the CONSTANS protein is subject to proteasomal degradation mediated by a ubiquitin ligase complex. It therefore requires the clock-controlled translation plus the stabilization by PHYA under long daylight to remain stable. CONSTANS can therefore be seen as an external coincidence sensor of both. In other words, it seems to play the role of the photoperiodic timer.

Once stabilized, CONSTANS binds to the promotor of the floral activator gene *FT* (*Flowering Locus T*) and activates it. FT is the long-sought florigen^11^. It is transported to the apical meristem in the shoot and activates flowering (Mathieu et al. 2007). The interaction of CONSTANS and FT is conserved among plants and the “CONSTANS/FT regulon” also functions in photoperiodic timekeeping of short-day plants, just the activity of CONSTANS-like proteins is regulated oppositely (Gendron and Staiger 2023).

Bünning was aware of the high light sensitivity of the photoperiodic responses in plants and developed the idea that the rhythmic leaf movements, also called sleep movements, have an adaptive significance during moonlit nights. When moonlight shines vertically on the upper epidermis of the leaves (the sensory organ for photoperiodic responses), it could trigger a long-day response (Bünning and Moser 1969). In short-day plants, moonlight could interfere with photoperiodic flower induction as they could misinterpret moonlit nights as long days and consequently flower less. By reducing the angle of exposure through sleep movements, the plants reduce the intensity of moonlight falling on the leaf surface to values between 5 and 20%, light intensities that do not trigger photoperiodic responses (Bünning and Moser 1969). Wolfgang Engelmann often referred to this study in his lectures. He explained that the main reason for the rhythmic leaf movements could be to avoid the disruptive effects of moonlight on photoperiodic responses. He also pointed out that the lowering of the leaves does not affect the entrainment of the circadian clock that controls rhythmic leaf movements, as its photoreceptors are in the leaf joints.

### Test of Bünning’s hypothesis on plants with “free-running” clocks

If the seasonal flowering response is indeed dependent on the circadian clock and is inhibited when light falls within a certain physiological state of the clock, it should be possible to inhibit flowering by providing light pulses to plants that are otherwise kept in constant darkness at precisely this phase but not at others. In other words, a series of light pulses administered to different plants under free-running conditions should rhythmically inhibit flowering approximately every 24 h when a light pulse hits the physiologically relevant phase of the clock. Again, such night interruption experiments can only be done in plants that survive several days in darkness, and Bünsow^12^ was the first to perform such experiments with white light in *K. blossfeldiana* (Bünsow 1953a, b). In fact, he found that white-light pulses of 2–4 h inhibited flowering approximately every 24 h, which supported Bünning’s hypothesis; but he also noticed that these light pulses shifted the phase of the circadian clock, as expected from the phase-response curve (Fig. 2).

**Fig. 4:**
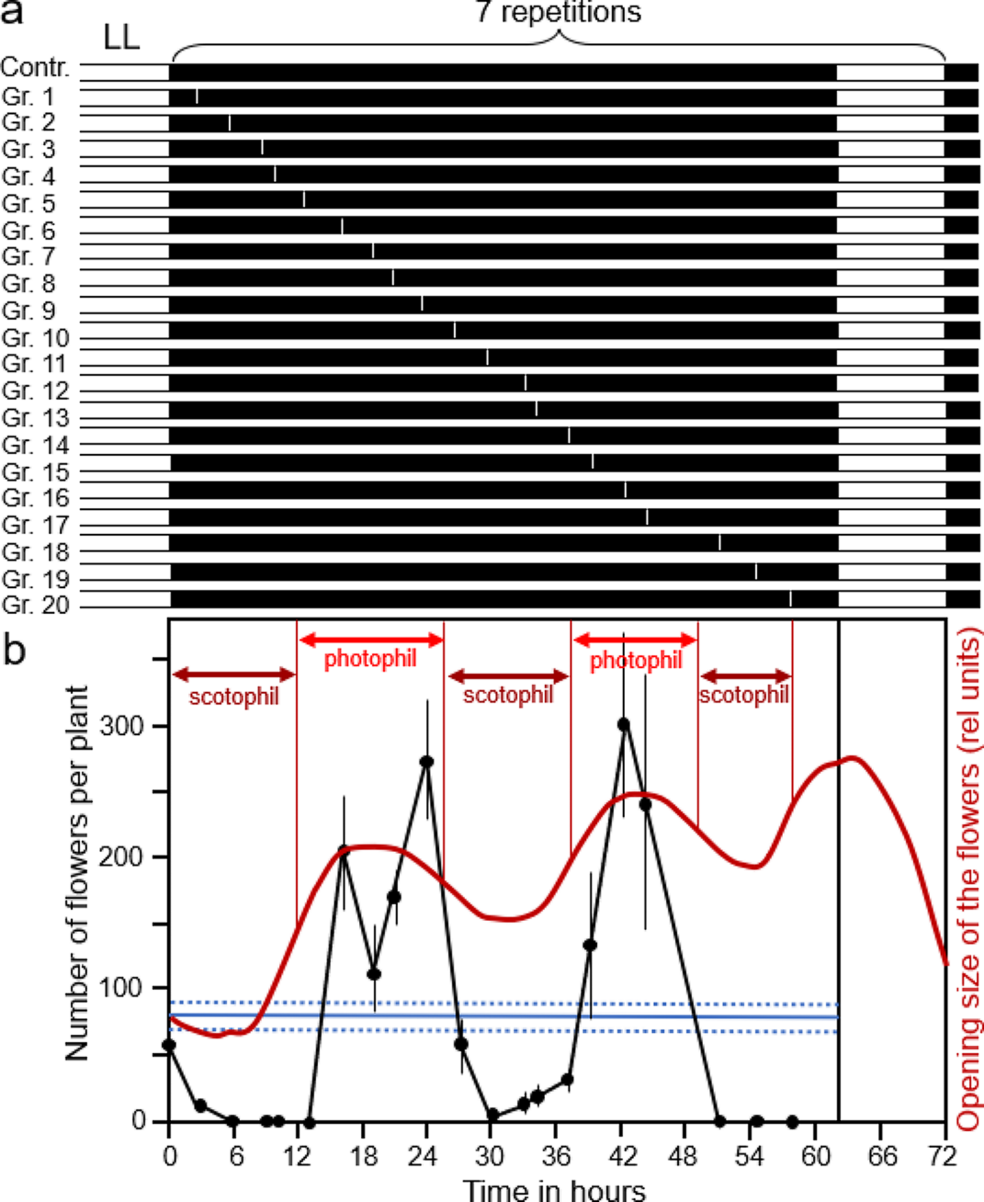
Dependence of flower formation on the time of red-light disturbance (2 min of 660 k erg cm^− 2^) during a 62 h dark period with a 72 h cycle that was applied seven times. **a** Light program for the controls and the 20 different experimental plant groups. The plants came from continuous light (LL) in the greenhouse and were placed back to LL after the seven 72 h cycles. The 2-minute red light pulses are indicated as thin white bars in the 62-hour dark period. **b** The number of flowers was counted 4 months after the end of the light induction. The blue line indicates the average number of flowers of the controls (± standard error of the mean, SE, as dotted blue lines). The number of flowers counted in the different groups of experimental plants is indicated as filled black dots (± SE) connected by black lines. The rhythm of petal movements is shown in red. The inhibition of flowering by red light roughly coincided with the assumed scotophil phase of the circadian clock (petals closed, see Fig. 2), while promotion of flowering by red light roughly coincided with its predicted photophil phase (petals open)

In his doctoral thesis, Wolfgang Engelmann tried to solve this problem by using red light (with a maximum at 655 nm) or far-red light (maximum at 733 nm) instead of white light. In preliminary experiments, he found that 2-minute-long pulses of red light with an energy of 660 kiloerg/cm^2^ were sufficient to inhibit flowering when applied in the scotophilic phase (Engelmann 1960). More importantly, by recording petal movements under the same conditions, he was able to show that these short pulses of red light did not phase-shift the circadian clock. In one of his main experiments, he used the following protocol, which was modified from Nanda and Hamner (1959). The plants were grown under continuous light in the green house until an age of 18 weeks, at which they are most sensitive for the induction of flowering by short days. Then, they were transferred into a climate chamber with constant temperature (20 °C) and constant humidity (80%) to start the short-day induction experiments. The control plants received seven cycles of 10 h light followed by 62 h of darkness (10 h:62 h light: dark cycles) before they were transferred back to constant light in the greenhouse. This treatment was known to reliably induce some flowering several weeks later (Melchers^13^,^1956^). The experimental plants received the same seven 10 h:62 h light: dark cycles, but each dark-phase was interrupted by one pulse of 2 min red light. This stimulus was given at different times to 20 different plant groups, each consisting of 4 plants (Fig. 3a). Four months after the end of the light induction, the number of flowers per plant was determined and averaged for each group of plants. Engelmann counted an average of 80 flowers per plant in the controls showing that the seven 10 h:62 h light-dark cycles induced flowering as was expected. In contrast, the red-light pulses antagonistically promoted and inhibited flowering (Fig. 4b). To find out whether the promotion and inhibition of flowering coincide with the photophil (petals open) and scotophil (petals closed) phases of the clock, he recorded the petal movements under the same environmental conditions (19 h:62 h light-dark cycles). Indeed, the cycles of flower induction run parallel to the endogenous circadian opening and closing movements of the flowers (red curve in Fig. 4b). These results strongly support Bünning’s model that the circadian clock is involved in the photoperiodic responses, and Bünning decided to publish them even before Engelmann’s dissertation in “*Die Naturwissenschaften*”^14^, a German scientific journal that was often used at the time for short reports on results of great general interest (Bünning and Engelmann 1960).

Engelmann’s results with far-red light pulses (733 nm), which were thought to inhibit flowering during the photophil phase but promote it during the photophil phase (Könitz 1958) were slightly different from what was expected. Far-red light pulses inhibited flowering not only during the photophil but also during the scotophil phase, which is hard to understand and indicates that the mechanisms by which the phytochromes regulate flowering are more complicated and still underexplored in short-day plants. As mentioned above, *Arabidopsis* possesses five phytochromes and only the active form of PHYA stabilizes CONSTANS. The active form of PHYB is mainly sensitive to far-red light and involved in CONSTANS degradation together with other factors (Song et al. 2015; Gendrom and Staiger 2023). To the author’s knowledge nothing is known about the phytochromes present in *Kalanchoë* and their detailed functions. Perhaps PHYB plays a similar role as in *Arabidopsis* and degrades CONSTANS when activated by far-red light. If true, this would explain the general inhibition of far-red light on flowering of *Kalanchoë.*

**Fig. 5:**
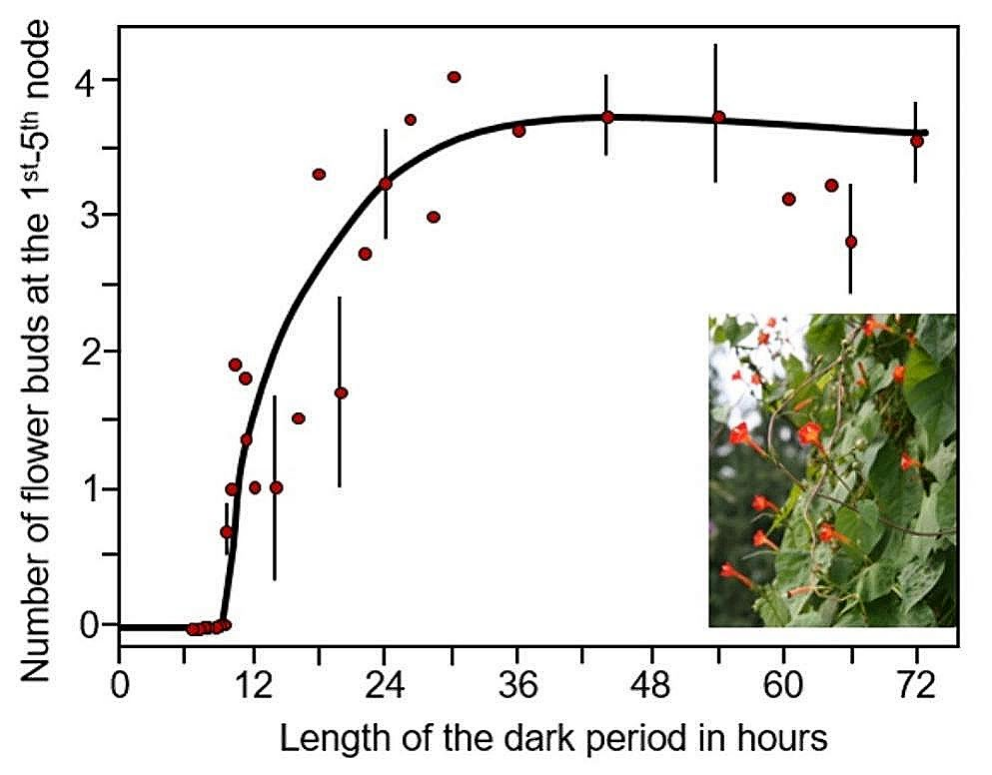
Dependence of flower formation on the length of a single dark period in *Ipomoea coccinea*. The points represent mean values of several (3–22, mostly about 10) plants. In some cases, vertical lines indicate the mean errors. Modified after Engelmann (1960). The picture of *I. coccinea* was taken by Michael Wolf in August 2008 in the botanical garden of Dresden

In another experiment, Engelmann applied a different Nanda-Hamner protocol and compared the rhythm of flower induction in *K. blossfeldiana* with the flower induction of another short-day plant, the Red Morning Glory (*I. coccinea*). *I. coccinea* is a fast-growing, twining, flowering climber that originates from tropical America and has been introduced to large parts of the USA. Like *K. blossfeldiana* it flowers between November and January. To test whether flower induction of *I. coccinea* relies also on a circadian clock, Engelmann kept the light phase at 10 h and systematically lengthened the dark phase from 6 h to 72 h. If controlled by a circadian mechanism, the photoperiodic response would only be triggered in a photoperiod that is a multiple of 24 h (e.g. light: dark cycles of 10 h:14 h; 10 h:38 h; 10 h:72 h) and not in non-24 h cycles (e.g. light: dark cycles of 10 h:20 h; 10 h:40 h). He found that in contrast to *Kalanchoë*, *Ipomoea* showed no rhythm in flower induction (Fig. 5). A single dark period that was longer than 10 h induced flowering, and flower induction became maximal after a dark period of 30 h. He concluded that *Ipomoea* does not employ a circadian clock but rather an hourglass clock for day length measuring (Engelmann 1960).

The hourglass clock of *I. coccinea* can also be explained by the phytochrome system. Once the dark period begins, the active Pfr form is slowly converted to the inactive Pr form, a process known as dark reversion. When enough Pfr has converted to Pr at a certain night length, flowering inhibition ends, and the plants begin to flower (discussed in Gendron and Staiger 2023).

## Summary and outlook

In summary, in his PhD thesis, Wolfgang Engelmann strongly confirmed Erwin Bünning’s theory that the circadian clock is involved in photoperiodism in the Flaming Katy, *K. blossfeldiana.* At the same time, he showed that flower induction in the Red Morning Glory, *I. coccinea* does not rely on a circadian clock, indicating that plants invented different mechanisms for seasonal adaptation. The same appears to be true for animals. Most but not all seem to rely on their circadian clocks to provide an internal reference for measuring day length (see articles in this issue). Bünning’s model of photoperiodism was later refined by Colin Pittendrigh and called the “External Coincidence Model” (Pittendrigh and Minis 1964; Pittendrigh 1966). The External Coincidence Model is in contrast to a second model, the “Internal Coincidence Model” (Pittendrigh and Daan 1976), according to which the organisms measure the phase relationship between two internal oscillators to determine day length (see Saunders 1978 and this issue, for a comprehensive overview). Both models rely on the circadian clock. A third model that works independently of the circadian clock and was briefly mentioned above is the “Hourglass Model”. It was originally developed for aphids (Lees 1960, 1966) and predicts that a hypothetical substance accumulates during darkness and as soon as this substance reaches a certain threshold, the photoperiodic reaction is triggered (Lees 1973). As outlined by Saunders and Lewis (1987), there is a smooth transition between oscillators and hourglasses, as oscillators can be of different strengths. Weak oscillators dampen quickly under constant conditions and become arrhythmic. This makes them difficult to distinguish from hourglasses. Several northern fly species have such weak (i.e., “damped”) clocks that do not continue for a long time under constant conditions, but nevertheless seem to use the External Coincidence Model for photoperiodic time measurement (Lankinen et al. 2021; Vaze et al. this issue). Lankinen et al. (2021) called this response quantitative external coincidence to distinguish from external coincidence of strong circadian clocks. Even in aphids photoperiodic time measurement appears to partly rely on a circadian clock (Colizzi et al. this issue) and similarly, the Japanese morning glory, *Ipomoea nil* (formerly *Pharbitis nil*), which is closely related to *I. coccinea* shows features of external coincidence (Lumsden and Furuya 1986). As modeled by Saunders and Lewis, there can be a smooth transition between external coincidence and hourglass suggesting that anything in between is possible in living systems. While conceptually the External and Internal Coincidence Models are distinct, it is very difficult with our currently available experimental approaches to distinguish between these mechanisms.

Nowadays, we also know that phytochromes are not the only photoreceptors involved in plant photoperiodic responses. Cryptochromes contribute to this process (Gendron and Staiger 2023; Song et al. 2015). Cryptochromes are UV-A/blue-light absorbing pigments that evolved from photolyases and are present in different forms in all organisms (reviewed in Deppisch et al. 2022; 2023). In plants, light activated cryptochrome inactivates the COP1/SPA complex (CONSTITUTIVE PHOTOMORPHOGENETIC/SUPPRESSOR OF PHYA complex) that promotes the degradation of CONSTANS (Yang et al. 2017). Therefore, plant cryptochrome allows CONSTANS to accumulate in the nucleus and activate the FT (florigen) gene. In animals, cryptochromes are mainly involved in the circadian clock itself or in entraining the circadian clock to light-dark cycles but they may additionally serve as photoreceptors for photoperiodic responses (e.g. Colizzi et al. this issue).

It is not surprising, that each species, and importantly, populations within species, as a consequence of mutation, natural selection, and genetic drift, can and do exhibit genetically-based local adaptation to the seasonal environment. Studying photoperiodic mechanisms in a range of different organisms will help to determine which mechanisms are conserved and which represent specific adaptations to a particular environment. Erwin Bünning and Wolfgang Engelmann were undoubtedly pioneers of chronobiology, and it remains exciting to follow in their footsteps.

## Epilogue

**Fig. 6:**
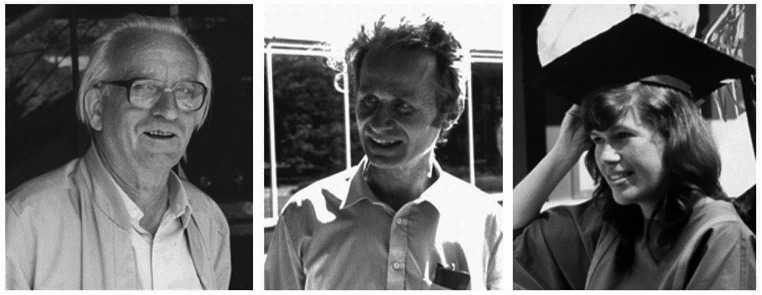
Three generations of chronobiologists at the Botanical Institute of the University of Tübingen (from left to right: Erwin Bünning, Wolfgang Engelmann, Charlotte Helfrich-Förster). These pictures were taken by Richard Helfrich on July 18, 1985, on CHF’s PhD defense

During my career, I was very fortunate to have Wolfgang Engelmann as my PhD supervisor and Erwin Bünning as my PhD grandfather. They gave me the intellectual background of chronobiology and taught me how to conduct scientific research. Wolfgang Engelmann also gave me the freedom I needed to develop my own ideas and carry out the experiments I thought would be useful. Whenever I needed advice, he was there to help me. Erwin Bünning was also still there regularly when I was doing my doctorate. I saw him almost every morning slowly climbing the stairs to the 2rd floor where he had his office. He was a taciturn man who exuded a great deal of authority, as was typical of German professors at the time. I rarely dared to speak to him, but when I did, he proved to be very open-minded. He seemed to know exactly what the Engelmann group was doing and kept a very close eye on our activities. Erwin Bünning was one of the examiners at my doctoral defense in 1985 (Fig. 6). He had a sharp mind and remained intellectually very active and up to date with the latest literature until he was 82 years old (1988), at which time he was hospitalized for pneumonia, which he survived but from which he never fully recovered (he died in 1990). I still remember the words of Wolfgang Engelmann that it is not good to save a life at any cost if that means prolonging it artificially when it is time to go. It was clear to me that Wolfgang Engelmann wanted a different ending for himself, but when it happened, it came far too suddenly. I called him on the evening of June 30, 2023, to ask about a book I needed to finish an article for this special issue. He didn’t have this book, but we immediately started talking about other scientific things. He told me about his plans to install a photovoltaic system on the roof of his house and that he had listened to several scientific lectures on the subject. We also talked briefly about his latest electronic book. As always, he was very animated and full of plans. It was a shock when his wife called me the next day to tell me that he had died on the way to a scientific meeting, probably of a pulmonary embolism. He was 89 years old, but it is still hard to believe that he is no longer with us. I miss him very much.

## Data Availability

No datasets were generated or analysed during the current study.

## Footnotes

1 Erwin Bünning (1906–1990) was a German biologist. He is considered a co-founder of chronobiology along with Jürgen Aschoff and Colin Pittendrigh. Bünning studied biology, chemistry, physics and philosophy at the Universities of Berlin and Göttingen and earned his Doctor of Philosophy from the University of Berlin in May 1929. In 1930, he took an assistantship under Otto Renner at the University of Jena, where he habilitated with research on the daily leaf movements of bean plants. In 1930, he had to leave Jena due to his anti-Nazi political convictions and accepted a teaching position at the East Prussian University in Königsberg. In 1938, Bünning spent a year traveling through Java and Sumatra. His observations on the islands resulted in the book *Tropische Regenwälder* (Tropical Rainforests). Upon his return to Germany in 1939, Bünning was conscripted in the German military (World War II). In 1942, military authorities appointed Bünning as an associate professor at the University of Strasbourg. After the war, Bünning became a full professor at the University of Cologne in 1945 before moving to the University of Tübingen the next year (1946), where he stayed until his retirement in 1971. Bünning published over 260 papers in the fields of plant physiology and general biology as well as a popular textbook on Plant Physiology and his famous book “The Physiological Clock” first in German (1958) and later translated into English (1964). He received many honors in his life both in the field of chronobiology and botany. A biography of Bünning’s life and scientific work in German can be found in Plesse (1996). In addition, there are several shorter tributes to him by his former students (Chandrashekaran 2006; Honegger et al. 2020).

2 Wolfgang Engelmann (1934–2023) was a German chronobiologist who investigated the rhythms of many different organisms. He studied biology at the University of Hamburg and the University of Tübingen. In 1960, he earned his doctorate with Erwin Bünning on the relation between the endogenous clock and the photoperiodic flower induction in two plant species. From 1961 to 1963 he spent a postdoc in David Shappirio’s lab at the University of Michigan extending his photoperiodic studies to a fly. In 1967, he spent a second postdoc with Colin Pittendrigh at the University of Princeton. Back at the University of Tübingen, he worked a long time as a research assistant, until he was appointed as a professor of biology in 1979. He remained in Tübingen until his retirement in 1999. Engelmann demonstrated the existence of endogenous clocks and characterized their properties in more than 20 different species. He published more than 100 articles including several online articles and books about biological rhythms (https://tobias-lib.uni-tuebingen.de/xmlui/browse?type=author&value=Engelmann%2 C+Wolfgang).

3 Colin Stephenson Pittendrigh (1918–1996) was a British-born biologist who spent most of his adult life in the United States. Pittendrigh is regarded as the “father of the biological clock,” and founded the modern field of chronobiology alongside Jürgen Aschoff and Erwin Bünning. He is known for his careful descriptions of the properties of the circadian clock in *Drosophila* and other species, and providing the first formal models of how circadian rhythms entrain (synchronize) to local light-dark cycles. He belongs to the most influential authors in J Comp Physiol A, and a more detailed curriculum vitae from him is published in Zupanc et al. (2024).

4 Kurt Stern (1904–1956) was a biochemist who obtained his doctorate in Berlin in 1930 and emigrated to Great Britain in 1933 and then to the USA. He was from 1944 to 1956 a professor for biochemistry at the Brooklyn Polytechnic Institute in New York.

5 Rose Stoppel (1874–1970) was a German botanist and plant physiologist. She was the first female professor of botany in Germany. She worked for 12 years as a domestic helper before she was allowed to study at the Universities of Berlin, Strasbourg, Freiburg, and Basel. She completed her doctorate on the influence of light on the opening and closing of several blossoms in 1910 at the University of Freiburg and habilitated 1924 at the University of Hamburg, where she became professor of botany in the same year. She continued to work on biological rhythms and made several important observations before she retired in 1944.

6 Wilhelm Pfeffer (1845–1920) was a German botanist who, together with Julius von Sachs, is considered the founder of modern plant physiology. Pfeffer studied at the Universities of Marburg and Berlin did his habilitation with Julius von Sachs on the effects of colored light on the decomposition of carbonic acid at the University of Würzburg. Pfeffer worked as a professor at the Universities of Bonn, Basel, Tübingen and Leipzig. He is famous for his work on osmosis and the development of a membrane osmometer, which was named “the Pfeffer cell” after him. Pfeffer investigated many other botanical phenomena, including the movement of the stamens of *Centaurea* species and chemotactic movements of spermatozoa in mosses and ferns, as well as the daily rhythms of leaf movements in *Phaseolus* and the starch saccharification of maize seedlings.

7 Anthonia Kleinhoonte (1887–1960) was a Dutch botanist and experimentalist who worked at the Botanical Laboratory in Delft, The Netherlands. She identified at least 50 plant species, but she is best known for her study of the circadian rhythms of plants. She created a device that measured jack bean (*Canavalia ensiformis*) leaf movements.

8 Ingeborg Beling (1904–1988) was the first scientist who examined the time memory of honeybees experimentally. In her doctoral thesis (Beling 1929), which she carried out under the supervision of Karl von Frisch, she systematically investigated the ability of honeybees to remember specific times of day at which they had been trained to appear at a feeding site. Beling can be regarded as a pioneer of chronobiology, as she described the basic characteristics of the circadian clock on which the honeybee’s memory for time is based. However, she did not call it a circadian clock, because at the time of her research the existence of circadian clocks was not yet recognized, and even in 1959 researchers in the von Frisch laboratory did not associate the bee’s time memory with a circadian clock (see Pittendrigh 1993. Page 27). After her PhD, Beling worked as only women among 64 scientists at the *Biologische Reichsanstalt* in Berlin. From 1929 to 1935 she published eleven papers on different aspects of insect biology before she ended her scientific career, probably due to the then enormous political pressure on married women to prioritize family over their careers. A review about her life and work can be found in Beer et al. (2024).

9 Rose Zimmer was a PhD student of Erwin Bünning who investigated the effects of light pulses on the circadian petal movements of *Kalanchoë blossfeldiana.*

10 Walter Könitz was a PhD student of Erwin Bünning. He investigated the flowering inhibition in short-day plants by light red and dark red light in the photophilic and scotophilic phase and published this work 1958 in *Planta*. In 1965, a second paper appeared in *Planta* with him as a sole author, in which he performed electron microscopic studies on *Euglena gracilis* in the diurnal light-dark cycle. This work was also done at the Botanical Institute of Tübingen.

11 The existence of a plant hormone called florigen (flower formation agent) that induces flowering was first demonstrated by the plant physiologist Mikhail Khristoforovich Chailakhyan in 1936. Chailakhyan conducted several sophisticated experiments that showed that the plants only flowered when the leaves were exposed to the critical day length. The daylength information is then transferred from the leaves to the shoot tips that trigger flower formation, suggesting that a substance travels a considerable distance from the leaves to the tip. Later, Chailakhyan proved that floral induction can be transferred by grafting from an induced plant to a plant that has not been induced to flower. Since this was possible even between different plant species, Chailakhyan assumed that florigen is of a general nature. For a long time, the nature of florigen was unknown and it was thought to consist of two components, gibberellins and anthesins. It was not until seventy years later that the existence of florigen in *Arabidopsis thaliana* was clearly demonstrated (overview in Aksenova et al. 2006).

12 Robert Bünsow (1919–2007) was a German Botanist, who worked at the Institute of Plant Physiology at the University of Göttingen in the 1950s and 1960s. He is best known for his light-interruption experiments that showed the participation of the circadian clock in photoperiodism.

13 Johann Georg Friedrich Melchers (1906–1997) was a German biologist. Melchers studied natural sciences at the Albert-Ludwigs-Universität Freiburg the Georg-August-Universität Göttingen, where he earned his doctorate in 1930. He then worked in Göttingen, Munich and Berlin. In 1945, he came to Tübingen, where he was professor at the Eberhard Karls University of Tübingen and from 1947 to 1976 director of the Max Planck Institute of Biology in Tübingen. He became fascinated by Bünning’s research and carried out several experiments in *Kalanchoë*. Otherwise, his research areas were heredity, plant viruses, evolution, and developmental physiology. He contributed to the elucidation of the genetic code of the tobacco mosaic virus. In the 1970s, he developed a new method for fusing plant cells to produce hybrids of closely related species.

14 In 1913, Arnold Berliner wanted to create a German counterpart to the English-language journal *Nature* with the journal “*Die Naturwissenschaften*”. In 1935, the Springer publishing house was forced to retire him because of his Jewish ancestry. No issue was published in 1945. The first post-war copy approved by the Allied Control Commission (under new management) was dated July 15, 1946. For many years, “*Die Naturwissenschaften*” was one of the most internationally respected journals. For example, the discovery of nuclear fission was published in the 6 January 1939 issue under the title “Über die bei der Bestrahlung des Urans mittels Neutronen entstehenden Erdalkalimetalle” by Otto Hahn and Fritz Straßmann. After 1945, the journal lost much of its importance. With an impact factor of 2.098 in 2014, it was well behind the Anglo-American journals Nature and Science but is still one of the leading international multidisciplinary journals.
